# Multifocal optical projection microscopy enables label-free 3D measurement of cardiomyocyte cluster contractility

**DOI:** 10.1038/s41598-023-46510-4

**Published:** 2023-11-13

**Authors:** Birhanu Belay, Edite Figueiras, Jari Hyttinen, Antti Ahola

**Affiliations:** 1 BioMediTech, Faculty of Medicine and Health Technology, Tampere University, Arvo Ylpön katu 34, 33520 Tampere, Finland; 2 Champalimaud Research, Champalimaud Centre for the Unknown, Lisbon, Portugal

**Keywords:** Cellular imaging, Microscopy, Heart stem cells, Biomedical engineering

## Abstract

Human induced pluripotent stem cell (hiPSC)-derived cardiomyocyte (CM) models have become an attractive tool for in vitro cardiac disease modeling and drug studies. These models are moving towards more complex three-dimensional microphysiological organ-on-chip systems. Label-free imaging-based techniques capable of quantifying contractility in 3D are needed, as traditional two-dimensional methods are ill-suited for 3D applications. Here, we developed multifocal (MF) optical projection microscopy (OPM) by integrating an electrically tunable lens to our in-house built optical projection tomography setup for extended depth of field brightfield imaging in CM clusters. We quantified cluster biomechanics by implementing our previously developed optical flow-based CM video analysis for MF-OPM. To demonstrate, we acquired and analyzed multiangle and multifocal projection videos of beating hiPSC-CM clusters in 3D hydrogel. We further quantified cluster contractility response to temperature and adrenaline and observed changes to beating rate and relaxation. Challenges emerge from light penetration and overlaying textures in larger clusters. However, our findings indicate that MF-OPM is suitable for contractility studies of 3D clusters. Thus, for the first time, MF-OPM is used in CM studies and hiPSC-CM 3D cluster contraction is quantified in multiple orientations and imaging planes.

## Introduction

Cardiovascular diseases represent one of the leading causes of mortality and morbidity globally^1^. Human induced pluripotent stem cell (hiPSC) derived cardiomyocytes (CMs) have provided a basis for in vitro studies on CM physiology, genetic cardiac diseases and on cardiotoxicity assays without using animal models^2,3^. Analyzing these cell cultures has prompted a need for developing novel analysis methods. Measurements such as patch clamp and microelectrode arrays have been used to extensively characterize their electrophysiology, but their biomechanical properties have not been characterized with much detail.

We have previously used video microscopy-based measurements that apply optical flow calculations to determine CM biomechanics^4–6^. Video microscopy analysis has provided a non-invasive, non-toxic, and low-instrumentation option for analyzing contractility. Since the development of hiPSC-CMs, numerous groups have applied these methods and it has become accepted as both a standalone measurement and as a method to augment other studies^7–9^. These methods have been used to analyze CMs as dissociated single cells^4,5,9^, monolayers^9,10^, and small clusters^11^. With the hiPSC-CM field maturing, there has been a drive for 3D structures involving beating CMs, and advances for performing analyses in 3D structures have been made^11,12^. The existing analysis methods, however, have been largely restricted to 2D space and with limitations to depth imaging in 3D. Thus, label-free imaging techniques capable of imaging samples in 3D are required for quantifying the magnitude and temporal parameters of contraction in CM clusters.

Fluorescence based imaging techniques such as selective plane illumination microscope (SPIM) can provide depth imaging with low phototoxicity. However, they still require fluorescent labeling, which could affect the cellular physiology^13^. On the other hand, light-field microscopy (LFM) has the ability to acquire 3D image data in a single shot and can be an essential tool to study cellular dynamics. However, it provides an image with reduced spatial resolution as compared to standard optical microscopy techniques^14^. In addition, 3D image reconstruction methodology in LFM is still under development^15^. Optical projection tomography (OPT) is a label-free, non-invasive high-resolution imaging technique capable of imaging 3D samples at higher penetration depth. It is an optical equivalent of X-ray micro-computed tomography. OPT has had various uses including imaging zebrafish embryo development^16,17^ and fibroblast cells in hydrogel scaffolds^18,19^. In standard OPT setups, different focal imaging of the sample is achieved by mechanically moving the objective lens or the sample, thus making the imaging of cardiac contraction unpractical. Implementing an electrically tunable lens (ETL) in an OPT setup enables extended depth of field without moving the sample. Further, ETL enables the focal shift in milliseconds, overcoming the acquisition speeds limits imposed by the mechanical movement. So far, ETL has been incorporated into imaging microscope setups for applications such as improving resolution and light collection efficiency in OPT^20^, upgrading 2D two-photon microscope for fast 3D imaging^21^, high speed imaging of beating zebrafish heart in SPIM^22^, and fast focusing in photoacoustic microscopy^23^. 3D imaging of CM clusters and assessing their contractility is still a largely unmet need, which can be addressed using ETL.

In this study, we aim to quantify 3D cardiomyocyte cluster contractility. For this, we developed multifocal optical projection microscopy (MF-OPM) by implementing ETL into the detection path of our in-house built OPT setup for multifocal imaging. We applied optical flow to quantify contraction in several imaging planes throughout large volume 3D CM-clusters in hydrogel. We demonstrate the measurement of large 3D CM clusters and show how MF-OPM and the contraction analysis can be used to image large 3D CM clusters in multiple focal planes and angles, how their contractility can be quantified. We measure the contraction and assess how the clusters can exhibit different contraction profiles for different focal planes and angles. Further, we demonstrate the setup by assessing the effect of temperature and adrenaline on contractility. To the best of our knowledge, this is the first use of MF-OPM in a CM study and the first 3D imaging-based quantification of CM cluster contraction dynamics.

## Materials and methods

### Ethical approval

The Ethics Committee of Pirkanmaa Hospital District has approved to conduct research on hiPSCs (R08070). The Heart Group, Tampere University (TAU), received the tissue biopsies from Tampere University Hospital, Tampere, Finland. All experiments were performed in accordance with relevant guidelines and regulations. Written informed consent was obtained from the donor and/or their legal guardian(s).

### Cardiomyocytes culture

CMs derived from hiPSCs using the Embryoid Body (EB) method were used in this study (Heart Group, TAU)^24^. The CM clusters were cultured in a flask with RPMI (Thermo Fisher Scientific, USA) supplemented with B27(+ insulin) (Thermo Fisher Scientific, USA) and 50 U mL^−1^ Pen/Strep. For 3D cell culturing, 2–4 CM clusters per cuvette (Sigma-Aldrich, USA) and perfluorinated ethylene propylene (FEP, Adtech Polymer Engineering, UK) tube was encapsulated in a commercial geltrex (Thermo Fisher Scientific, USA) hydrogel and incubated with an EB-medium. The EB-medium consisting of KnockOut-DMEM medium (Gibco Invitrogen, USA) supplemented with 20% fetal bovine serum (FBS) (Gibco Invitrogen, USA), 1% non-essential amino acids (Cambrex BioSciences, Verviers, Belgium), 1% L-glutamine (Invitrogen, USA), and 50 U/ml penicillin/streptomycin (Cambrex BioSciences, Vertviers, Belgium).

### Sample preparation

The CM cluster-hydrogel samples were prepared in an optically transparent glass cuvette for ease of administration of drugs post baseline experiment (adrenaline) or in FEP tube for imaging for rotation-based experiments. The diameter of the cuvette in the direction of optical path is 5 mm. FEP tube has an inside diameter of 1.5 mm. For cuvette-based experiments, 500 µl of geltrex hydrogel per cuvette was prepared. The cardiomyocyte clusters were picked up using a pipette and inserted into a hydrogel in non-gelated form. For FEP experiments, the mixture of liquid hydrogel and CMs were inserted into the FEP tube. After the encapsulation of the CM clusters in a hydrogel, the samples were let to gelate in the incubator for 10 min at 37 °C. Then, the EB medium was added to the top of the CMs-hydrogel sample. The samples were incubated at humidified 37 °C in 5% CO_2_. The cell medium was changed every three days, and the cells were cultured for seven days before MF-OPM imaging.

### Multifocal optical projection microscopy system

**Figure 1:**
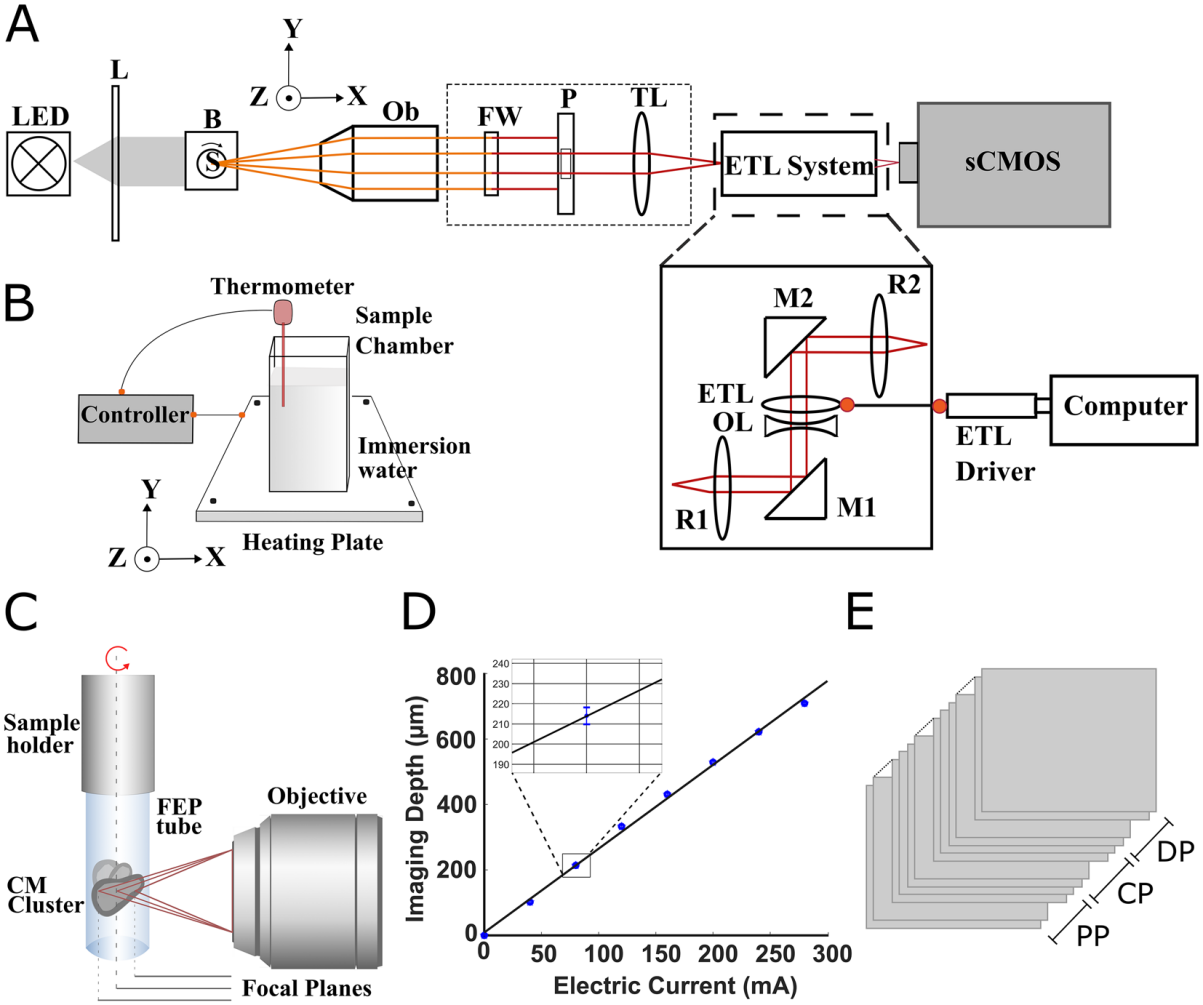
Schematics of multifocal-optical projection microscopy (MF-OPM) imaging setup. (**A**) The ETL system incorporated between tube lens and sCMOS camera. The ETL lens together with offset lens (OL) positioned horizontally between two relay lenses (R1 and R2) and two mirrors (M1 and M2). The CM-hydrogel cluster sample inserted in FEP tube and immersed in refractive index matching water bath (B) and rotation stage (S). The transmitted light from light emitting diode (LED) through telecentric lens (L) is collected by an infinity corrected objective lens (Ob), is passed through the filter wheel (FW) and a pinhole (P), tube lens (TL), and electrically tunable lens (ETL), and is detected by sCMOS camera. (**B**) The temperature control consists of heating plate (attached the MF-OPM sample X–Y stage), thermometer (inserted in water bath), and controller. (**C**) Illustration of multifocal imaging in MF-OPM. (**D**) The relationship between the applied electric current to ETL and image depth for 10x/0.28 NA microscopic objective in MF-OPM. (**E**) The grouping of focal planes to proximal (PP), central (CP) and distal (DP) planes obtained using multifocal imaging.

The MF-OPM imaging system is based on integrating a remotely controlled fast focus and electrically tunable lens (ETL) paired with an offset lens (f_offset_ = -150 mm) (EL-20-30-Ci-VIS-LD-MV, Optotune AG, Switzerland) in the detection path of in-house built OPT setup^19,25^ (Fig. 1). The ETL system was placed between the tube lens (f_tube_ = 200 mm) (Mitutoyo, USA) and the sCMOS camera (ORCA-Flash 4.0, Hamamatsu, Japan), as shown in Fig. 1A. In this configuration, the ETL was positioned between two relay lenses (optical relay 4f system, f = 100 mm) (Achromatic doublet lens, Thorlabs, USA) at the conjugate back focal plane of the detection lens. The optical relay system was used to control the change in image magnification across the Z-range raised by the ETL. Since ETL is a liquid lens, it was mounted horizontally. Two mirrors (M1 and M2, Thorlabs, USA) were used to guide the light into the ETL. Lens driver 4i (Optotune AG, Switzerland), with a control range of 0–300 mA, was used to control the ETL. By remotely controlling the ETL, focusing on the desired position was achieved in milliseconds without moving the sample or objective lens.

The samples prepared in a cuvette or FEP tube were submerged in a larger cuvette, the sample chamber, filled with water (refractive index-matched liquid). MF-OPM system can be operated in both brightfield and fluorescence imaging modes. For brightfield imaging, a white light-emitting diode (LED) source with telecentric lens was used to illuminate the sample. The transmitted and emitted light were detected either by a 10x/numerical aperture (NA) 0.28 or 5x/NA 0.14 infinity-corrected microscope objective (Ob, Edmund, USA) and imaged with an sCMOS camera. The center of rotation was manually aligned using a motorized linear x–y–z stage (Standa, Lithuania). The multifocal images were acquired by applying different current values into the ETL. The MF-OPM system is controlled using in-house-developed LabView-based (National Instruments, USA) image acquisition software.

To perform living cell experiments, we also incorporated an in-house-developed temperature control system into the MF-OPM setup (see Fig. 1B). The system consists of a heating plate (Zerodis, China) and a temperature control unit with a thermometer (Inkbird, China). The heating plate was attached to the MF-OPM x–y–z stage. The sample chamber filled with water was placed in the heating plate and heated at 37 °C, followed by the submersion of the sample. The temperature of the sample was monitored/controlled during the experiment using feedback obtained from a temperature sensor (thermometer) submerged inside the water bath.

### ETL calibration

We calibrated ETL current with respect to focal depth using fluorescent beads with a diameter of 15 µm, embedded in 1% agarose gel inside a cuvette. We focused on a single bead at a time using a 10 × microscope objective. Then, the position of the focal plane was varied (as shown in Fig. 1C) by applying an electric current to ETL (0–280 mA) at an interval of 40 mA. With each change in an electric current, the shift in-depth distance was measured by moving back to the original focus position (center of the bead) along optical axis using the motorized linear stage (as shown in Fig. 1D). The distance moved by the linear stage is measured and used to generate the calibration curve. The measurement was repeated four times using different beads positioned at different locations in the field of view to generate ETL current vs mean depth with standard deviation plot.

### Cardiomyocyte imaging

#### Temperature experiment

We quantified the CM beating behavior response to change in temperature. The beating of hydrogel-embedded CMs, prepared in cuvettes, were recorded using MF-OPM with temperature control. The video image data was acquired using a 10 × microscope objective with a NA of 0.28, focusing in the middle plane of each cluster. First, the sample in cuvette was kept at 37 °C in the water bath for 10 min (as shown in Fig. 1B). Then, the heating was stopped, and the controlled temperature drop was monitored using the thermometer. The beating of the CM clusters was recorded at the rate of 20 or 50 frames per second (FPS) for 20 s, repeated for each temperature drop of 2 °C (36.8–24.8 °C).

#### 3D imaging experiment

The CM cluster contractility profiles from multiple planes and angles was measured by preparing two CM-hydrogel samples in FEP tubes. The schematic demonstrating multiangle and multifocal imaging of CMs in MF-OPM is shown in Fig. 1C. The samples were mounted in a motorized rotation stage. The temperature of the samples was kept constant at 37 °C. The contraction video data was acquired at 0°, 45°, and 90° using MF-OPM (10x/NA 0.28 objective lens). The CM contraction was recorded at 21 focal planes for each angle at constant exposure time. Videos were recorded at 20/50 FPS for 20 s, for a total of approximately 21 min. Additionally, we visualized and quantified the shape of a CM cluster by obtaining projection images for eight focal planes over 360° with a rotational step size of 0.9° for 3D image reconstruction.

#### Adrenaline experiment

The effect of adrenaline on CM contractility was evaluated by preparing three CM-hydrogel samples in a cuvette. (±)-Epinephrine hydrochloride (adrenaline, Sigma-Aldrich, USA) was dissolved in Milli-Q water for final concentration of 1 µM. First, baseline (BL) videos were recorded before administering the adrenaline. The adrenaline solution was then pipetted on top of the CM-hydrogel sample. To enable adrenaline (ADR) to diffuse and reach the CM cluster, we waited for 20–30 min before video acquisition. In this experiment, the video of CM contraction was recorded at a single projection angle (0°) from 21 focal planes with a rate of 20/50 FPS for 20 s, for a total of approximately 7 min. The temperature of the sample was kept at 37 °C. The images were acquired using 10x/NA 0.28 microscope objective. The size of a single frame was 2048 × 2048 pixels with a pixel size of 0.65 µm × 0.65 µm.

### Contraction analysis in 3D

For each imaging plane and angle, velocity vector fields of the contractions were first calculated from the video recordings as reported previously^6^, i.e. displacement relative to the previous frame using minimum quadratic difference based dense optical flow calculation, filtering of spurious vectors and defining directional beating signals using beating focus points. A representative region of the cluster with visible cellular structures was selected to generate a directional beating signal, and key timepoints of a contraction cycle were determined: contraction start, contraction end, relaxation start and relaxation end^6^. These timepoints were defined from the contraction and relaxation peaks in the beating signal as follows: Three-sample windowed variance of the signal from the peak towards nearest relaxed state was calculated and normalized. Then, cumulative trapezoidal integral of the resulting variance signal was calculated, forming the integrated variance signal. We defined the end-of-motion using the 95% of the maximum of integrated variance signal as a threshold value, producing the contraction start and relaxation end timepoints. Contraction end and relaxation start points were defined from the minimum of the squared transient between contraction and relaxation peaks.

In addition to the previously reported method, here a second round of calculation was performed to determine the displacement for each contraction cycle with respect to the reference frame, indicated by the start of contraction. As reported previously^6^, dense optical flow was calculated using a 32 × 32 window with 50% window overlap and 150 pixel (px) maximum displacement. Outlier vectors were removed and replaced by cubic spline interpolation, and in the case of resulting spurious vectors, again by linear interpolation. The measured displacement in brightfield was validated using fluorescent bead displacement, as described in Supplementary Materials section Validation of Displacement and Supplementary Fig. S1. For each individual plane, these displacements were synchronized by determining the time point of 1 px displacement, and the contraction cycles were averaged to calculate a single waveform from five beats. This extra step was done to counteract possible mismatch of timing in 20 FPS recordings.

For each plane, a mean contraction waveform from multiple beats was generated. Due to the limited number of measurement points, a cubic spline was fitted to this waveform. This was then compared with the other imaging planes/angles for temporal characteristics. Magnitude characteristics were calculated from unfitted data points. For visualization and comparison between the clusters, the 21 imaging planes were grouped into three groups, representing proximal (1–7), central (8–15) and distal (16–21) layers, as indicated in Fig. 1E. Further, we determined the similarity of the image plane properties by quantifying the image intensity and measured image 2D Fast Fourier transform (FFT) from planes 1, 11 and 21.

### Image processing and reconstruction

Due to the size of the clusters, we extended the depth of field by all-in-focus stacking from multifocal brightfield projection images using Stack Focuser fusion algorithm (Umorin M, available online https://imagej.nih.gov/ij/plugins/stack-focuser.html). Then, a standard filtered back-projection algorithm was applied to reconstruct a 3D image from stack-focused images for 3D visualization and quantification of size and shape parameters. All the computation were performed in MATLAB (Mathworks, USA) environment. The 3D reconstructed image was further processed using Avizo (Thermo Fisher Scientific, USA) software. The 3D image was first inverted for better visualization. Next, the CM cluster was segmented from the background by manually thresholding via adjusting the minimum and maximum gray values. Finally, we used the Avizo label analysis module to quantify the cluster aspect ratio by ellipse fitting and volume by adding up the voxel elements. The aspect ratio was defined as the ratio of medium to largest axes, with elongated cluster shape represented by small axes ratio.

## Results

### Imaging performance of electrically tunable lens

We investigated the performance of ETL for depth imaging in MF-OPM. To do this, we determined the relationship between the applied electric current in the ETL and the shift in the image focal plane. This relation is shown in Fig. 1D. We found that the applied current in ETL was linearly related to shift in-depth in MF-OPM. Similarly, the same tunable lens (calibration data from Optotune AG, Switzerland) showed the linear relationship between the ETL refractive power and applied current (Supplementary Fig. S2). Thus, the result demonstrates the suitability of ETL for depth imaging in MF-OPM imaging microscope.

### Effect of temperature on cardiomyocyte beating behavior

**Figure 2:**
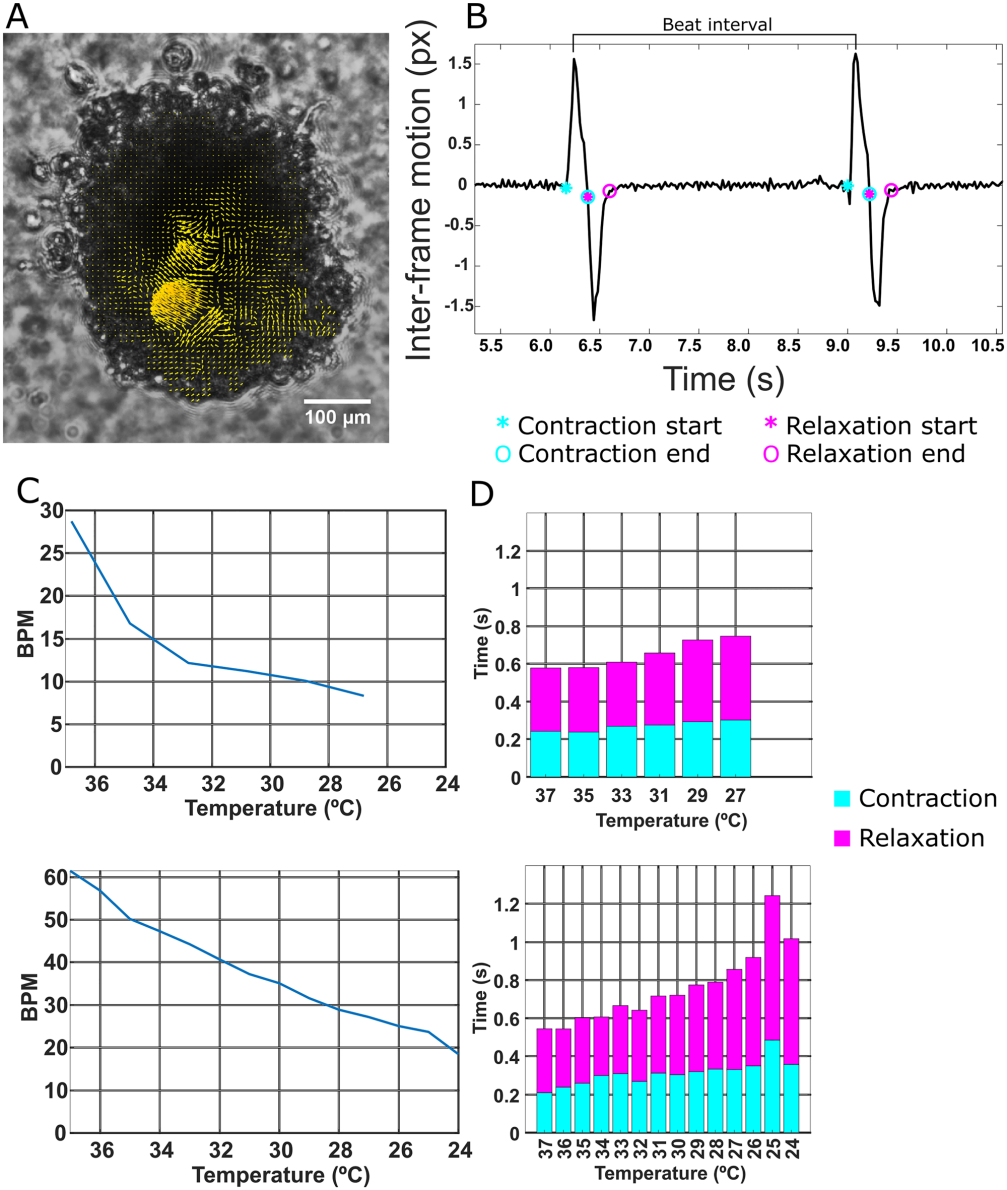
The effect of temperature on CM contraction characteristics. (**A**) The displacement vector field of a CM cluster shows the largest displacements during the contraction at 37 °C. (**B**) The parametrization of contraction and relaxation start and end points from velocity signal measured between subsequent video frames, with contraction-associated points in cyan and relaxation in magenta. Beats per minute (BPM) calculated from the beat interval defined as 60 s per duration between contraction peaks. (**C**) BPM as a function of temperature for both clusters, showing the decline when decreasing from 37 °C to 24 or 26 °C. (**D**) The durations of contraction and relaxation as a function of temperature.

In experiments with living cells in vitro, controlling the temperature of the cell sample is vital in maintaining the functionality of the cells. We aimed to determine the effect of temperature change on the beating behavior of iPSC-CM clusters. For this, we performed video recordings of two contracting clusters with an MF-OPM imaging microscope while decreasing the temperature. The representative brightfield projection image of a CM cluster co-registered with displacement vector field of CMs contraction recorded at 36.8 °C, is shown in Fig. 2A. The contraction and relaxation motion is parametrized and shown here in Fig. 2B, as described previously^4^. Here, the sum of contraction and relaxation associated motion is indicated as mechanical activity. The displacement vector field represents the magnitude and direction of CM contraction in the cluster, as shown in Fig. 2A. The mean beating frequency as beats per minute (BPM) of the cluster decreased with the temperature in both clusters (28–8 BPM at 36.8–26.8 °C, and 61–18 BPM at 37–24 °C) (Fig. 2C). The decrease in temperature increased the relaxation duration and to lesser extent, the contraction duration (Fig. 2D). For one of the clusters, contraction was too weak to analyze below 26.8 °C. These results confirm the effect of temperature variation in CM contraction dynamics. Thus, this underlines the importance of a temperature-controlled environment in iPSC-CMs experiments.

### Three-dimensional analysis of cardiomyocyte contractility

**Figure 3:**
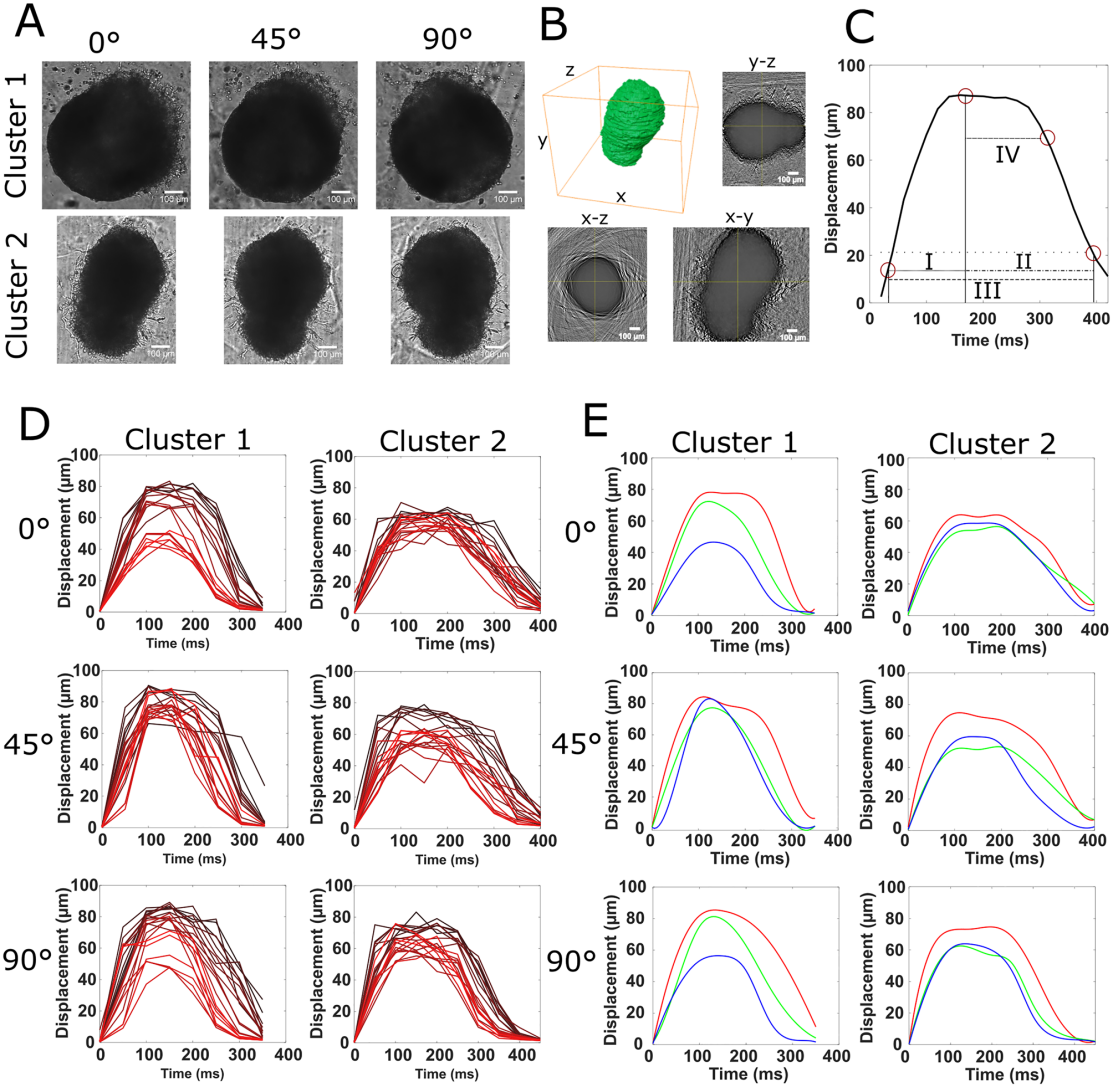
The characterization of CM clusters in 3D. (**A**) OPM projection images of CM clusters at 0°, 45° and 90° orientations. (**B**) The 3D reconstruction of cluster 2 and its orthoslices. (**C**) The parametrization of a contraction transient: (I) contraction phase, (II) relaxation phase, (III) total mechanical activity and (IV) contracted phase. The circles indicate, from left to right, onset of contraction, transient peak maximum, 75% of the peak maximum and 25% of the peak maximum. (**D**) Individual contraction traces from clusters 1 and 2 for each rotation. The red shading of each trace shows their plane position, with proximal planes are shown in darker red whereas distal focal planes lighter red. (**E**) Spline fitted and averaged waveforms based on focal group for clusters 1 and 2. Red indicates proximal, green central and blue distal focal plane groups.

We aimed to determine if the image projection angle and depth affect the measured contractility. This was done by acquiring multifocal contraction video data using three distinct imaging angles (0°, 45°, 90°) using MF-OPM, producing 63 videos per cluster (21 planes for each angle). Here, we used two clusters, shown in Fig. 3A and Supplementary Videos 1 and 2. Cluster 1 had a round shape while cluster 2 had a more elongated form. A 3D reconstruction of cluster 2 was performed as described in “Image processing and reconstruction” section, shown in Supplementary Video 3. From the reconstruction (Fig. 3B), we measured its volume to be 6.8 × 10^–7^ µm^3^ and its elliptical aspect ratio 0.4 (medium/major). The quantified contraction transients were parametrized as shown in Fig. 3C, as contraction (I)—from start to peak, relaxation (II)—from peak to 25% of peak maximum, total mechanical activity (III)—from start to 25% of peak maximum, and the shape ratio, defined as the ratio of (III) and (IV)—from peak to 75% of peak maximum.

The contractile characteristics of these two clusters with respect to angle and imaging depth were quantified. The imaging planes were grouped to proximal, central and distal planes (PP, CP and DP) as previously explained in “Contraction analysis in 3D” section. Individual contraction traces from these planes and the resulting PP, CP and DP traces are shown in Fig. 3D. The contractile parameters from the groups are shown in Table 1.

**Table 1:** Measured contraction parameters and standard deviations from the rotation experiment.

Rotation	Cluster 1			Cluster 2		
	0°	45°	90°	0°	45°	90°
Beats per minute	20 ± 4	20 ± 3	20 ± 4	58 ± 5	62 ± 5	64 ± 5
Contraction (ms)						
PP	155 ± 35	115 ± 15	150 ± 24	171 ± 35	106 ± 22	160 ± 58
CP	122 ± 20	120 ± 19	117 ± 15	163 ± 40	149 ± 64	134 ± 40
DP	128 ± 21	120 ± 36	128 ± 35	152 ± 31	128 ± 43	158 ± 41
Relaxation (ms)						
PP	140 ± 29	187 ± 27	179 ± 29	177 ± 32	240 ± 38	187 ± 53
CP	136 ± 23	136 ± 25	158 ± 39	201 ± 46	199 ± 47	179 ± 26
DP	118 ± 22	113 ± 34	108 ± 26	178 ± 33	158 ± 49	138 ± 41
Total mechanical activity (ms)						
PP	296 ± 17	302 ± 20	329 ± 17	349 ± 20	346 ± 22	347 ± 7
CP	258 ± 24	256 ± 20	274 ± 46	364 ± 13	348 ± 27	313 ± 22
DP	246 ± 8	233 ± 33	237 ± 12	329 ± 24	287 ± 16	295 ± 10
Transient shape ratio						
PP	30%	44%	29%	30%	48%	33%
CP	30%	30%	28%	21%	30%	36%
DP	26%	23%	25%	29%	29%	24%
Magnitude (µm)						
PP	77.6	83.7	84.9	53.5	74.4	74.6
CP	69.6	75.7	79.5	56.1	53.1	61.2
DP	45.9	78.5	56.3	58.5	59.5	63.6
Mean magnitude (µm)	64.4	79.3	73.6	56.0	62.3	66.5

We further compared the temporal characteristics of each plane group in cluster 1 to an individual plane from the center of the same cluster (plane 11) at 45° rotation, as if measured using traditional single-plane video brightfield microscopy. We found the contraction time to differ from 111 to 148%, relaxation time from 70 to 121% and total mechanical activity from 90 to 127%.

Within the cluster, BPM remained similar at each orientation. The contraction magnitude varied at different depths in the round-shaped cluster 1, whereas in the less symmetric cluster 2 the orientation was a more prominent factor, as shown in Table 1 and Fig. 3D,E. The image property results (Supplementary Materials section Image properties) showed that mean intensities and intensity range do not explain the variation in magnitude. Our 2D Fourier transform (FFT) results (Supplementary Table S2 and Fig. S3) showed that the differences in focal plane frequency content did not explain the difference in CM contraction magnitude either. The largest differences were observed between the central plane and proximal/distal plane in nearly all clusters.

Regarding temporal parameters, the contraction transient shapes were different in both clusters with respect to cluster orientation, while the shapes were similar between depths, as shown in Fig. 3D,E. Thus, the results reveal that MF-OPM-based video analysis captures contraction characteristics, which are different with respect to depth and orientation in 3D CM clusters.

### Effect of adrenaline on cardiomyocyte contractility

**Figure 4:**
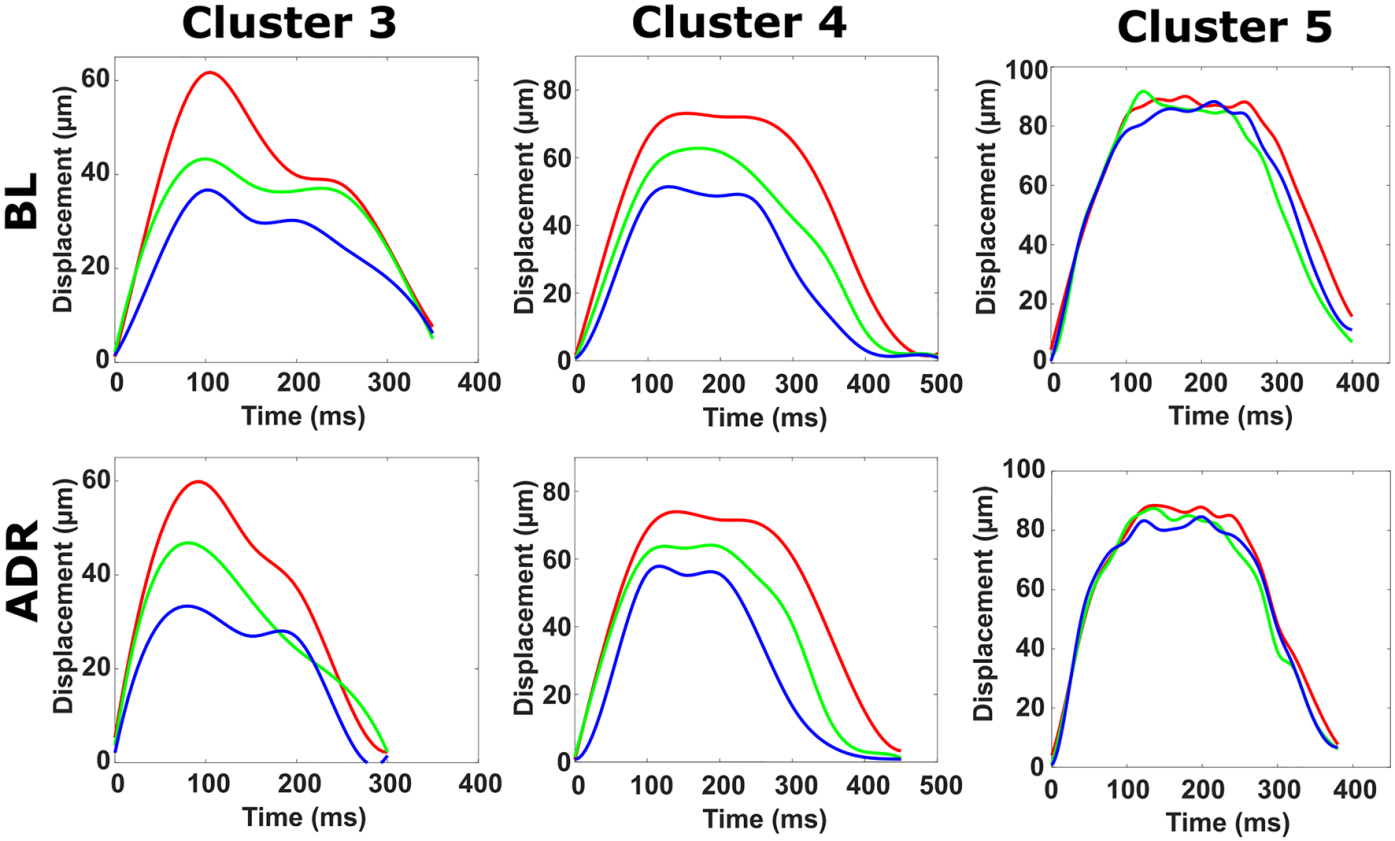
Averaged waveforms of clusters 3, 4 and 5 from the focal groups in baseline (BL) and adrenaline (ADR) measurements, each group being an average of multiple focal planes: red represents proximal, green central and blue distal focal groups. Clusters 3 and 4 were recorded with 20 FPS, and cluster 5 with 50 FPS.

We measured the effect of adrenaline on the contractility of CM clusters in different imaging planes imaged using MF-OPM. Three clusters were imaged from one direction in 21 imaging planes prior (baseline) and after administering adrenaline, as shown in Supplementary Video 4. Supplementary Fig. S4 illustrates the contractility in the 21 focal planes in baseline. The imaging planes were grouped as described before in “Contraction analysis in 3D” section. Adrenaline had a discernible effect in all three clusters as shown in Table 2: adrenaline increased BPM in all clusters, with the change ranging from 20 to 50%. Temporal parameters related to relaxation phase decreased, but no clear effect was seen in the duration of contractile motion. The overall mechanical activity decreased 19–25%, 8–14% and 6–8% for clusters 3–5, respectively, depending on focal group (PP, CP and DP). No notable changes in magnitude of contraction in the presence of adrenaline were observed when compared to baseline. The averaged signals from the focal groups are shown in Fig. 4. These results suggest that adrenaline affects the beating rate and the duration of relaxation associated motion.

**Table 2:** Measured contraction parameters and standard deviations from the baseline (BL) and adrenaline (ADR) experiment.

	Cluster 3		Cluster 4		Cluster 5	
	BL	ADR	BL	ADR	BL	ADR
Beats per minute	94 ± 30	141 ± 7	51 ± 2	62 ± 5	22 ± 2	35 ± 2
Contraction (ms)						
PP	103 ± 18	102 ± 44	199 ± 60	139 ± 27	183 ± 47	197 ± 48
CP	147 ± 82	82 ± 20	177 ± 38	161 ± 43	134 ± 17	137 ± 28
DP	130 ± 49	134 ± 58	155 ± 45	135 ± 43	171 ± 48	168 ± 48
Relaxation (ms)						
PP	216 ± 23	146 ± 36	201 ± 64	246 ± 24	197 ± 37	147 ± 52
CP	179 ± 79	181 ± 21	191 ± 35	176 ± 44	215 ± 25	195 ± 22
DP	202 ± 51	108 ± 51	180 ± 53	161 ± 30	188 ± 43	164 ± 45
Total mechanical activity (ms)						
PP	319 ± 24	248 ± 16	399 ± 13	385 ± 28	380 ± 13	345 ± 14
CP	326 ± 19	263 ± 23	368 ± 13	338 ± 5	349 ± 11	331 ± 16
DP	332 ± 23	243 ± 12	335 ± 15	296 ± 13	359 ± 15	332 ± 7
Transient shape ratio						
PP	20%	23%	30%	44%	31%	21%
CP	30%	22%	26%	28%	39%	36%
DP	25%	17%	29%	30%	33%	29%
Magnitude (µm)						
PP	61.6	59.4	73.1	73.8	90.0	88.4
CP	43.3	43.4	62.4	63.7	91.6	87.2
DP	36.7	32.2	50.5	55.8	88.1	84.6

## Discussion

We developed label-free multifocal optical projection microscopy (MF-OPM) -based imaging and contractility assessment tools of cardiomyocyte (CM) clusters. This enabled us to quantify the contractile activity of 3D CM clusters inside 3D hydrogel culture. CM clusters are inherently three-dimensional and measuring their contraction using 2D methods represents an ill-posed problem. By integrating an electrically tunable lens (ETL) into our in-house-built standard optical projection tomography (OPT) setup^19,25^ and tuning the imaging and optical flow methods accordingly, we were able to characterize for the first time CM cluster contractility in 3D. We demonstrated the imaging and quantification procedure by measuring the contractile activity of clusters in different orientations, focal planes, temperatures and in the presence of adrenaline.

Our ETL system showed a clear linear relationship between the change in focal position (depth) and applied electric current. This enabled multifocal imaging, capable of generating evenly spaced z-stacks, representing different layers of the cluster. Using ETL, we achieved extended depth imaging up to 700 µm, as opposed to being limited to traditional microscope objective from a single focal plane (here, 10–15 µm with 10x/NA 0.24), not optimal for thick cell samples. Without using the ETL, mechanically moving the sample for depth imaging is constrained by the working distance of the microscopy objective. Moreover, this could perturb the sample, especially for studies involving dynamic biological processes. Here, ETL enabled the depth imaging of large 3D CM clusters, not possible using a standard OPM setup. This underlines the importance of ETL in CM contractility studies. The setup further incorporated a temperature control unit. We demonstrated it and quantified the beating of CM clusters while decreasing the temperature. As in previous studies, temperature decrease resulted in a decreased beating rate and we observed similar results regarding the mechanical activity duration as shown before^26^. We confirmed that the beating behavior of CMs is influenced by the temperature. Thus, the developed setup enabled a temperature-controlled environment for 3D cell culture studies and assessment of contractility.

In this work, the developed technology enabled us to characterize the contractility of these clusters using the developed MF-OPM by quantifying the motion in different angles and imaging planes, resulting in a representation of contraction in 3D. Our results indicate that the shape, size, and orientation of clusters affect the contraction behavior. This was seen in the magnitude of contraction within the same clusters in different orientations as the difference in mean contraction magnitude was approximately 20%. This indicates that the cluster orientation is critical in contractility measurements of CM clusters. We also observed temporal differences between depths, with shorter durations of contraction observed in distal as compared to proximal positions. Cluster orientation had a smaller effect on the duration of mechanical activity as compared to depth in cluster, underlining the importance of ETL in an MF-OPM setup in this application. Beating rate remained unaffected by the orientation and depth. Comparing the measurements from the grouped focal planes to a measurement from a single plane from the center of the cluster further revealed the notable differences in all measured parameters. Together, these findings suggest that contractility is captured differently depending on orientation and depth in a 3D cluster. Thus, the study shows the importance of the method in quantifying the 3D aspects in video microscopy-based contraction measurements.

Finally, we demonstrated the developed MF-OPM setup by measuring the adrenaline response to CM contractility. We found adrenaline to decrease the duration of relaxation and increase the BPM by approximately 50% in two of the clusters and 30% in one cluster. However, we observed adrenaline to have no clear effect on the magnitude of contraction. While adrenaline is a known positive inotrope, conflicting results on magnitude of contraction have been reported previously for human induced pluripotent stem cell derived cardiomyocytes (hiPSC-CMs), further depending on the beating rate and cell line^27,28^. To our knowledge, the response of hiPSC-CM clusters of this size to adrenaline has not been reported. This measurement showed that the method can give insight to the 3D contraction, providing magnitude and duration parameters of the beating behaviour.

Further, our multifocal results revealed large inter-plane variation in contraction magnitude. Our results revealed that neither image intensity nor frequency content of the image planes explained the differences in contraction magnitude. The FFT analysis showed proximal and distal planes to have similar frequency content, as opposed to the central plane. This effect could be due to scattering, influenced by cluster size and cell density distribution. Together, these findings suggest that the observed differences in contractility are stemming from the cell cluster itself. Therefore, ignoring the inter-plane variation as done in 2D microscopy of 3D clusters could be misleading. This underlines the importance of 3D analysis for 3D CM cluster drug studies, as introduced here.

The presented MF-OPM setup enabled the measurement of contractility in multiple layers and orientations. Beating in large CM clusters is inherently 3D, and the quantification of beating using optical flow would be best characterized by a true 3D vector field. The method presented here measures motion on a stack of 2D planes separately, without taking cross-plane motion into consideration. In a future study, this cross-plane motion could be measured by synchronizing the acquisition of images by pacing the cluster either electrically or optically. Thus, optical flow could be measured in adjacent imaging planes as well. This could lead to methods with full 3D visualization of contraction. An alternative approach could involve integrating the concept of light-field microscopy into MF-OPM setup to capture CM contractility in true 3D from multiple projection angles. By detecting and fusing the light-field from multiangle, this method has the potential to improve image resolution and reconstruction^29^.

The quantification of motion relies on visible textures within the cluster. This is influenced by the light attenuation, introducing differences in quantification of motion for different sized clusters. The penetration of light through the inner structures of large and dense clusters requires long exposure times, reducing the frame rate. In this work, we imaged a CM cluster with a diameter of approximately 700 µm (i.e. the maximum focal distance shift using ETL with 10 × objective lens in the presented setup) with a maximum exposure time of 50 ms per frame. This could be improved further by using a light source in the near-infrared region with deeper light penetration, providing more visible textures at the cost of resolution. This would also enable lower light exposure time, resulting higher imaging frame rates, important for quantifying the temporal dynamics in disease models.

## Conclusion

We developed MF-OPM by incorporating ETL into our in-house built OPT setup and combined it with optical flow-based contraction analysis optimized for 3D quantification. ETL enabled extended depth of field imaging of large clusters, which is not possible using traditional microscope objectives without moving the sample or the objective lens. We used the developed method to quantify contractility of 3D CM clusters in a temperature-controlled environment. The MF-OPM with temperature control allows live imaging of CM clusters in a prolonged measurement, necessary for 3D imaging.

We demonstrated the capability of MF-OPM by analyzing large size CM clusters at different orientations and depths, in the presence of adrenaline and at different temperatures. We observed adrenaline to decrease CM relaxation duration and increase the beating rate. Our findings also revealed the variation in magnitude of contraction and duration of mechanical activity at different depths and cluster orientations. Thus, using 2D optical flow measurement to study 3D CM clusters can be misleading due to differences in cluster shape and orientation. These findings call for caution in video-based measurements of cardiac cluster contractility using 2D methods and recommend further 3D method development for this purpose using optical methods.

Here, we presented for the first time an advanced label-free 3D optical imaging method combined with contraction analysis for in vitro quantification of CM cluster biomechanics. The method can be further applied for applications in organ-on-chip studies involving disease models, drug development and personalized medicine.

### Supplementary Information

Supplementary Information 1.

Supplementary Video 1.

Supplementary Video 2.

Supplementary Video 3.

Supplementary Video 4.

## Data Availability

The datasets generated and analyzed in the study are available from the corresponding author upon reasonable request.
