# Supplementary material for: Multifocal optical projection microscopy enables label-free 3D measurement of cardiomyocyte cluster contractility
Source: Sci Rep. 2023 Nov 13;13:19788. doi: 10.1038/s41598-023-46510-4 (PMC10643565; doi:10.1038/s41598-023-46510-4)
Supplement: Supplementary file 1 — Supplementary Information 1. [file 41598_2023_46510_MOESM1_ESM.pdf]

Supplementary material

Multifocal Optical Projection Microscopy Enables Label-Free 3D Measurement of Cardiomyocyte Cluster Contractility

Birhanu Belay [1, *], Edite Figueiras [2], Jari Hyttinen [1], Antti Ahola [1,*]

[1] BioMediTech, Faculty of Medicine and Health Technology, Tampere University, Arvo Ylpön katu 34, 33520, Tampere, Finland.

2 Champalimaud Research, Champalimaud Centre for the Unknown, Lisbon, Portugal

*Email: birhanu.belay@tuni.fi, antti.ahola@tuni.fi

Validation of Displacement

For fluorescence video recording, the CM cluster expressing mScarlet red cultured together with 4 µm diameter spherical fluorescence beads in hydrogel. The sample was illuminated in epifluorescence mode in MF-OPM setup using an LED with an excitation wavelength of 530/33 nm, and emitted light with emission bandpass filter of $\lambda = 578 \pm 16$ nm was detected by a 10x/numerical aperture (NA) 0.28 infinity-corrected microscope objective (Ob, Edmund, USA) and imaged with an sCMOS camera. The sample also imaged in brightfield mode using MF-OPM with an imaging parameter like stated in the method section of the main article. The contraction video data was acquired at 20 frames per second for a total of 20 seconds.

We validated the displacement calculation by comparing the displacement of fluorescent beads in fluorescent video imaging to the displacement in brightfield video between a CM cluster relaxed and a contracted state, as shown in Supplementary Fig. 3. The fluorescent beads displacement was measured using ImageJ TrackMate [1], and the displacement in brightfield was measured using the presented method. The region of interest in brightfield was defined as a 25 px wide band from the boundary of the segmented cluster. The measured median displacement was 8.3 ± 2.8 px for fluorescent beads, and 9.9 ± 2.0 px for the textures in brightfield imaging. Pixel (px) size of 0.65 µm x 0.65 µm.

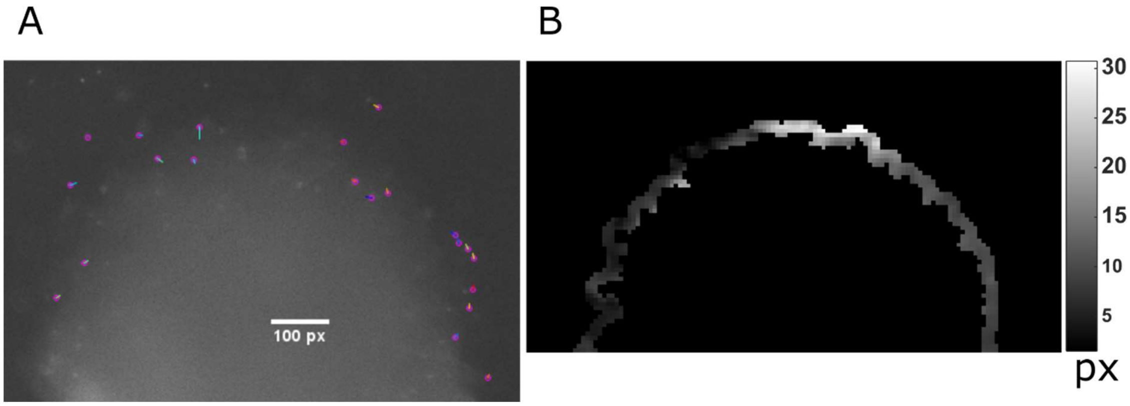

Supplementary Figure S1. Displacement validation from fluorescent beads. A) Displacement of fluorescent beads at the boundary of the CM cluster obtained from MF-OPM fluorescence imaging B) Displacement at the boundary of the CM cluster measured from MF-OPM brightfield imaging.

ETL Focal Power

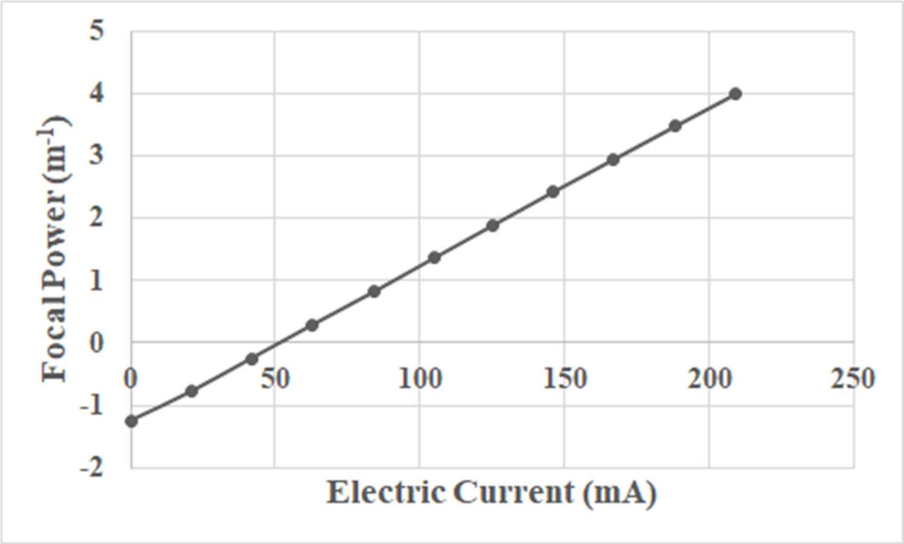

Supplementary Figure S2. The refractive power of electrically tunable lens (ETL) (EL-20-30-Ci-VIS-LD-MV) as a function of applied electric current (measured by the Optotune AG).

Image Properties

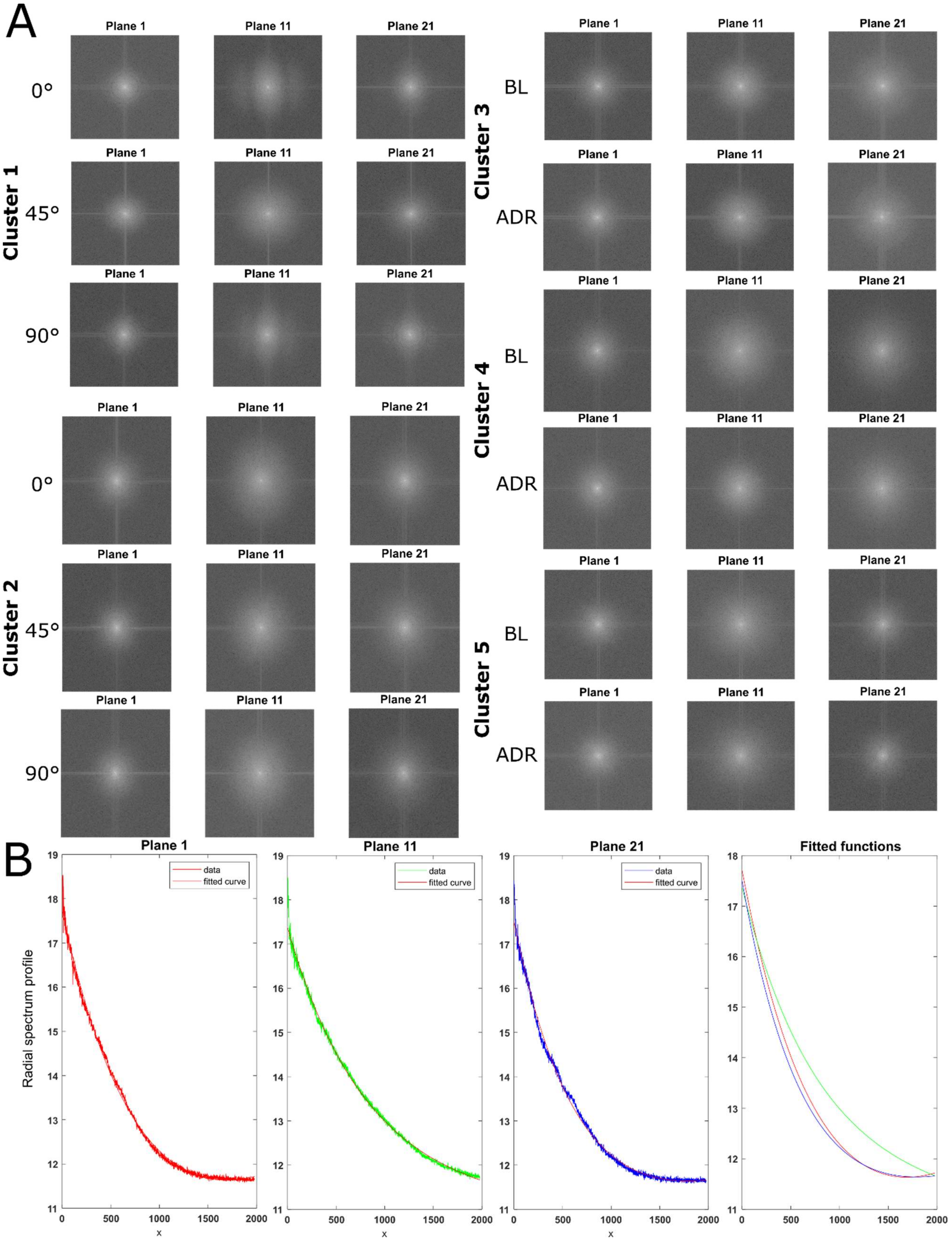

Supplementary Figure S3. Image frequency analysis. A) The 2D Fourier transforms (FFT) of image planes 1, 11 and 21 in Clusters 1-5, with rotations 0, 45 and 90 degrees for Clusters 1-2, and baseline (BL) and adrenaline (ADR) for Clusters 3-5. B) The radial spectrum profiles of the FFT images from Cluster 1 at planes 1, 11 and 21, illustrating the fitting of the exponential function to the image data.

Supplementary Table S1. The image minimum, mean and maximum intensities in imaging planes 1, 11 and 21 in Clusters 1-5, with rotations 0, 45 and 90 degrees for Clusters 1-2, and baseline (BL) and adrenaline (ADR) for Clusters 3-5.

	MIN			MEAN			MAX		
	Plane 1	Plane 11	Plane 21	Plane 1	Plane 11	Plane 21	Plane 1	Plane 11	Plane 21
Cluster 1, 0°	4868	3982	3476	8191	8535	8585	25898	65535	46011
Cluster 1, 45°	5154	4298	3960	7592	7573	7422	26119	65535	36910
Cluster 1, 90°	4834	3920	3594	7780	7885	7824	30208	61329	41832
Cluster 2, 0°	5278	4913	5135	9673	9655	11088	36544	65535	48623
Cluster 2, 45°	5966	5150	4802	9728	9757	11123	31920	65535	65535
Cluster 2, 90°	6084	5525	5081	9765	10155	11536	36249	65535	65535
Cluster 3, BL	4289	3436	3437	7338	7361	8210	21534	40267	54596
Cluster 3, ADR	4127	3325	3207	7234	7426	8501	20361	52576	65535
Cluster 4, BL	3096	2367	2150	5821	6210	6281	17543	65535	65535
Cluster 4, ADR	2844	2104	1815	5266	5360	5916	17702	26979	65535
Cluster 5, BL	1547	1706	1710	2654	3133	3148	8522	51807	14933
Cluster 5, ADR	1820	1683	1668	3042	3114	3175	9819	33231	12697

Supplementary Table S2. The calculated absolute area between focal planes (1, 11, 21) from FFT radial spectrum profiles fitted with 2nd order exponential curves. For function fitting, minimum R^2 = 0.989 and maximum R^2 = 0.9970.

	Radial FFT profile difference		
	1-11	1-21	11-21
Cluster 1, 0°	1228	292	998
Cluster 1, 45°	1436	364	1171
Cluster 1, 90°	920	205	808
Cluster 2, 0°	1579	293	1305
Cluster 2, 45°	1183	646	551
Cluster 2, 90°	1450	547	925
Cluster 3, BL	530	494	275
Cluster 3, ADR	713	599	311
Cluster 4, BL	1207	1126	275
Cluster 4, ADR	573	941	441
Cluster 5, BL	1083	129	1080
Cluster 5, ADR	858	236	1030

Displacement Profiles in Focal Planes

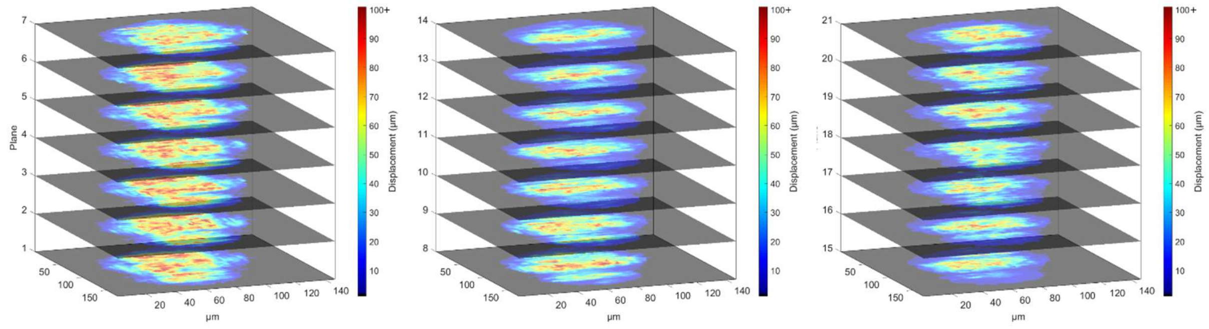

Supplementary Figure S4. The magnitude of contraction measured from cluster 2 (45 degrees) at each plane, represented as stacks of heatmaps.

Supplementary video materials

Supplementary Video S1: Beating of a CM cluster in brightfield shown in three angles (0°, 45° and 90°), synchronized to the first contraction.

Supplementary Video S2: Beating of a CM cluster in brightfield shown in three imaging planes (1, 11 and 21), synchronized to the first contraction.

Supplementary Video S3: Three-dimension reconstruction image of cardiomyocyte cluster in transparent hydrogel from brightfield imaging.

Supplementary Video S4: Beating of a CM cluster in brightfield before and after administering adrenaline, synchronized to the first contraction.

References:

1. Tinevez, J.-Y. *et al.* TrackMate: An open and extensible platform for single-particle tracking. *Methods (San Diego, Calif.)* **115**, 80–90 (2017).
